# Distinguishing Tumor Admixed in a Radiation Necrosis (RN) Background: ^1^H and ^2^H MR With a Novel Mouse Brain-Tumor/RN Model

**DOI:** 10.3389/fonc.2022.885480

**Published:** 2022-05-30

**Authors:** Xia Ge, Kyu-Ho Song, John A. Engelbach, Liya Yuan, Feng Gao, Sonika Dahiya, Keith M. Rich, Joseph J. H. Ackerman, Joel R. Garbow

**Affiliations:** ^1^ Department of Radiology, Washington University, Saint Louis, MO, United States; ^2^ Department of Neurosurgery, Washington University, Saint Louis, MO, United States; ^3^ Department of Surgery, Washington University, Saint Louis, MO, United States; ^4^ Division of Neuropathology, Department of Pathology and Immunology, Washington University, Saint Louis, MO, United States; ^5^ Alvin J. Siteman Cancer Center, Washington University, Saint Louis, MO, United States; ^6^ Department of Internal Medicine, Washington University, Saint Louis, MO, United States; ^7^ Department of Chemistry, Washington University, Saint Louis, MO, United States

**Keywords:** MRI, tumor, radiation necrosis, metabolic imaging, deuterium

## Abstract

**Purpose:**

Distinguishing radiation necrosis (RN) from recurrent tumor remains a vexing clinical problem with important health-care consequences for neuro-oncology patients. Here, mouse models of pure tumor, pure RN, and admixed RN/tumor are employed to evaluate hydrogen (^1^H) and deuterium (^2^H) magnetic resonance methods for distinguishing RN vs. tumor. Furthermore, proof-of-principle, range-finding deuterium (^2^H) metabolic magnetic resonance is employed to assess glycolytic signatures distinguishing RN vs. tumor.

**Materials and Methods:**

A pipeline of common quantitative ^1^H MRI contrasts, including an improved magnetization transfer ratio (MTR) sequence, and ^2^H magnetic resonance spectroscopy (MRS) following administration of ^2^H-labeled glucose, was applied to C57BL/6 mouse models of the following: (i) late time-to-onset RN, occurring 4–5 weeks post focal 50-Gy (50% isodose) Gamma Knife irradiation to the left cerebral hemisphere, (ii) glioblastoma, growing ~18–24 days post implantation of 50,000 mouse GL261 tumor cells into the left cerebral hemisphere, and (iii) mixed model, with GL261 tumor growing within a region of radiation necrosis (^1^H MRI only). Control C57BL/6 mice were also examined by ^2^H metabolic magnetic resonance.

**Results:**

Differences in quantitative ^1^H MRI parametric values of R1, R2, ADC, and MTR comparing pure tumor vs. pure RN were all highly statistically significant. Differences in these parameter values and DCE_AUC_ for tumor vs. RN in the mixed model (tumor growing in an RN background) are also all significant, demonstrating that these contrasts—in particular, MTR—can effectively distinguish tumor vs. RN. Additionally, quantitative ^2^H MRS showed a highly statistically significant dominance of aerobic glycolysis (glucose ➔ lactate; fermentation, Warburg effect) in the tumor vs. oxidative respiration (glucose ➔ TCA cycle) in the RN and control brain.

**Conclusions:**

These findings, employing a pipeline of quantitative ^1^H MRI contrasts and ^2^H MRS following administration of ^2^H-labeled glucose, suggest a pathway for substantially improving the discrimination of tumor vs. RN in the clinic.

## Introduction

Most neuro-oncology patients, including those with primary or secondary malignant brain tumors, are treated with radiation as an early therapeutic modality. These patients are at risk of developing delayed adverse effects related to the prior radiation, including *late time-to-onset* radiation necrosis (RN). Additionally, patients are at risk of developing recurrent cancer, most often within regions of the previously irradiated brain. Changes in post-therapy surveillance magnetic resonance imaging (MRI) features due to RN alone or recurrent tumor admixed with RN often present overlapping RN vs. tumor signatures. Thus, post-treatment MRI may be indeterminate and unable to accurately distinguish changes dominated by RN from recurrent tumors growing within an area of RN. While MRI multi-parametric analysis, including diffusion-weighted and dynamic susceptibility contrast (DSC) MRI, has improved differentiation of RN from mixed recurrent tumor/RN, standard, quantitative MR metrics for distinguishing these pathologies are still lacking (1–4). Similar indeterminacy is evident from ^1^H MRS studies (5–8) in which various endogenous metabolite metrics show 70–80% sensitivity and specificity in distinguishing RN from recurrent tumors.

Thus, the problem of accurately identifying tumor recurrence within a background of RN distinct from pure RN remains an important unmet clinical need in the management of neuro-oncology patients. Identification of imaging biomarkers capable of accurately distinguishing lesions dominated by RN from recurrent tumors admixed within regions of RN would be a major clinical advance, allowing oncologists to offer the best available treatment options in a timely fashion.

For many years, we have studied the late effects of radiation on normal brain tissue, having developed and characterized extensively a mouse model of late time-to-onset RN (9–18). Recently, we have described the late persisting impact of previous radiation on subsequent tumor growth (11) and on the responsiveness of tumors to immunotherapy (12). Of late, we have employed this radiation-biology platform to develop a novel mouse model that recapitulates many of the clinical features of recurrent malignant gliomas. In this murine model, tumor cells that have *not* been exposed to radiation are implanted within the ipsilateral hemisphere ~3.5–4 weeks after Gamma Knife (GK) irradiation of 30 or 50 Gy. The morphology of lesions in this post-irradiation implantation model is strikingly distinct from lesions resulting from gliomas implanted into non-irradiated brains. These findings are robust and have been observed in two syngeneic orthotopic murine malignant glioma models in two different mouse strains: i) DBT cells implanted in Balb/c mice (11) and ii) GL261 cells implanted in C57BL/6 mice (12).

As noted above, while distinguishing radiation necrosis from recurrent tumors remains a vexing clinical problem with important health-care consequences for neuro-oncology patients, there are obvious research limitations to the recruitment of relevant clinical populations for development and validation of new imaging biomarkers. A major challenge is the lack of quantitative correlative histology, which is not readily available in most patients and is, at best, highly localized tissue obtained using invasive surgical procedures (re limited biopsy or craniotomy with surgical resection). Our mouse models offer specific platforms with stratified populations of pure RN, pure tumor, and tumor growing in a RN background, the “mixed” model. Further, hematoxylin and eosin (H&E) histology provides a “gold standard” confirmation of lesion location/boundary and, in the mixed model (RN invaded with tumor cells), lesion identity. These animal models allow us to test the robust, standard-of-care imaging capability of MR biomarkers, whether structural, functional, or metabolic. The goals of this study were to employ our mouse models of pure tumor, pure RN, and admixed RN/tumor to evaluate a pipeline of common quantitative hydrogen (^1^H) MR contrasts, including magnetization transfer ratio (MTR), for distinguishing RN vs. tumor. The MTR contrast—tumor vs. RN—was improved over previous work from this laboratory (16) by the selection of pulse sequence parameters that minimized direct saturation of the ^1^H water signal. Further, proof-of-principle, range-finding deuterium (^2^H) metabolic magnetic resonance was employed following administration of ^2^H-labeled glucose to assess glycolytic signatures, distinguishing RN vs. tumor.

## Materials and Methods

### Mouse Models

#### General

All experiments were performed in accordance with the guidelines of Washington University’s Institutional Animal Care and Use Committee and were approved by that committee. Seven-to eight-week-old female C57BL/6 mice (Envigo Laboratories; Indianapolis, IN), housed four or five per cage in a light- and temperature-controlled facility, were used in this study. Three mouse models were studied: i) pure RN, ii) pure GL261 tumor, and iii) a “mixed” model, with tumor growing in RN. Separate cohorts of mice were employed in ^1^H MRI and ^2^H MR spectroscopy experiments.

#### Gamma Knife Irradiation

All mouse-brain irradiations were performed using the Leksell GK Perfexion™ (Elekta, Stockholm, Sweden), a device used for stereotactic radiosurgery of patients. Mice were anesthetized with a mixture of ketamine (25 mg/kg) and xylazine (5 mg/kg), injected intraperitoneally (IP), and restrained on a custom-built platform mounted to the GK’s stereotactic frame. Single-fraction, 50-Gy radiation doses (50% isodose), generated using the GK’s 4-mm collimator, were focused on the left cortex at a site ~2 mm posterior to the bregma. Irradiated mice developed RN beginning approximately four weeks post-irradiation, as described previously (15).

#### Tumor Implantation

For tumor implantation, mice were anesthetized with isoflurane and secured in a stereotactic head holder. Murine GL261 glioblastoma cells were implanted (~50,000 cells suspended in 10 μl per mouse) over 3 min at a site coincident with the focus of the GK radiation, 2 mm posterior and 3 mm to the left of bregma, and 2 mm below the cortical surface. For the pure tumor model, GL261 cells were implanted into non-irradiated mice. In the mixed model, GL261 cells were implanted into the irradiated hemisphere four weeks post irradiation.

### ^1^H MRI

#### Experimental Setup

The ^1^H MRI experiments employed a 4.7-T small-animal MR scanner with an Agilent/Varian (Santa Clara, CA) DirectDrive™ console and an Oxford Instruments (Oxford, United Kingdom) horizontal superconducting magnet. Data were collected with a laboratory-built, actively-decoupled transmit and receive RF coil pair: a 7.5-cm ID volume transmitter coil and a 1.5-cm OD surface receiver coil. Mice were placed on a warm-water pad and anesthetized with isoflurane/O_2_ (1.2–1.5% isoflurane) throughout the experiment. During MRI procedures, a Small Animal Instruments (SAI, Stony Brook, NY) monitoring and gating unit was used to monitor mouse respiratory rate, ~60 breaths/min, and body temperature, 37 ± 0.5 °C (rectal probe). Before placing each mouse into the magnet, a tail-vein catheter was inserted for subsequent injection of Gd-based contrast agent (CA): 100 µl 50% (V/V) Dotarem^®^ (Guerbet LLC USA, Princeton, NJ) in saline.

#### ^1^H MRI Pipeline

**Figure 1:**
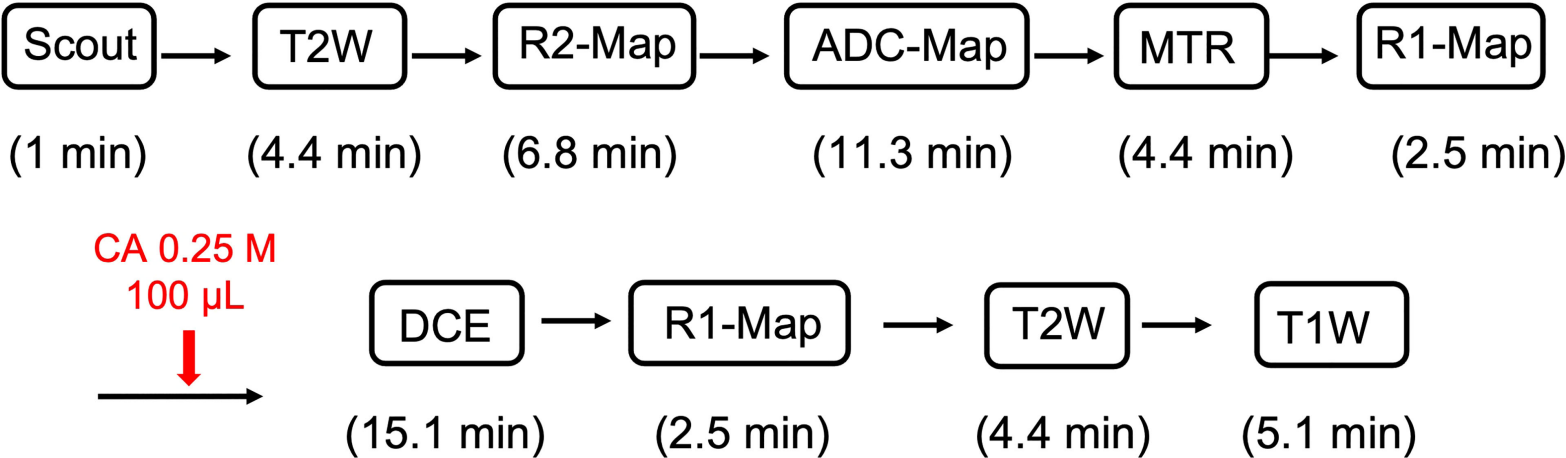
Schematic of the ^1^H MRI data acquisition pipeline.

A multi-contrast pipeline (**Figure 1**) was applied to models of pure RN, pure tumor, and tumor growing in RN. The parameters associated with all of the pipeline pulse sequences are listed below. Unless otherwise noted, matrix size was 64 × 64, field of view (FOV) 16 × 16 mm^2^, and 21 slices with a slice thickness of 0.5 mm.

#### T2-Weighted Imaging

T2W images were acquired with a 2D fast spin-echo multi-slice (FSEMS) sequence: echo train length (ETL) 4, Kzero 4, TR 2 s, effective TE 52 ms; matrix size 128 × 128, 4 averages.

#### T1-Weighted Imaging

T1W images were acquired with a 2D spin-echo multi-slice (SEMS) sequence: TR 0.4 s, effective TE 11 ms, matrix size 128 × 128, 4 averages.

#### R1 Mapping

R1 maps were acquired using a 2D gradient-echo multi-slice (GEMS) sequence: TR 0.1 s, TE 1.6 ms; flip angles of 5, 10, 15, 30, 50, and 70 degrees; 4 averages.

#### R2 Mapping

R2 maps were acquired with a 2D multi-echo multi-slice (MEMS) sequence: TR 6.0 s, echo spacing 11 ms in 16 steps (11 ms to 176 ms); 1 average.

#### Diffusion Weighted Imaging

Diffusion-sensitive images (re ADC) were acquired with a 2D FSEMS sequence: ETL 2, Kzero 1, TR 1.5 s, effective TE 30 ms, the duration of diffusion sensitizing gradient lobe (δ) 3 ms, time between diffusion sensitizing gradient lobes (Δ) 18 ms; 6-direction diffusion encoding (19), max b-value 1,000 s/mm^2^, 2 averages.

#### Magnetization Transfer

Magnetization transfer data were acquired using a 2D FSEMS sequence with the RF irradiation used to induce magnetization transfer turned either on or off: ETL 8, Kzero 1, TR 2.0 s, effective TE 6.5 ms, and 8 averages following 4 full dummy scans to establish a steady state. Magnetization transfer RF irradiation: 10-ms Gaussian-shaped pulses (flip angle 900 degrees) offset 2,000 Hz (10 ppm) from the water resonance. The parameter values were chosen to minimize the direct saturation of the ^1^H water signal.

#### Dynamic Contrast Enhancement (DCE)

In concert with bolus tail-vein administration of CA, DCE data were acquired with a GEMS sequence: 15 slices (center slice co-registered to the center slice of the 21-slice array), TR 47 ms, TE 1.6 ms, 1 average, flip angle 30°, and 300 time frames with 3-s time resolution.

### ^2^H MR Spectroscopy

#### Experimental Setup

The deuterium metabolic magnetic resonance experiments were designed as proof-of-principle, range-finding studies to discern the ability of the technique to discriminate pure tumor from pure RN. For these initial ^2^H experiments, we employed single-voxel spectroscopy (SPECIAL) performed at 11.74 T with an Agilent/Varian DirectDrive™ console and an Agilent/Magnex (Yarnton, Oxford, United Kingdom) horizontal superconducting magnet. ^1^H images, used for field shimming, planning, and anatomic registration of the ^2^H data, were collected with a 50-mm ID volume coil tuned to 499.3 MHz. ^2^H RF transmission and reception employ a one-turn 21-mm ID ^2^H surface coil tuned to 76.65 MHz. Anesthesia and physiologic monitoring were as described above for ^1^H MR imaging experiments.

#### Semiheavy Water Natural Abundance

Quantitative ^2^H MRS benefits from knowledge of the local (regional) natural abundance of ^2^H in the water supply, as this will also be the natural-abundance ^2^H concentration in tissue water (*vide infra*), which provides a convenient internal concentration reference. Titration experiments using the method of standard addition were employed to establish/confirm the ^2^H natural abundance in the St. Louis region water supply (20). This measurement of ^2^H natural abundance is directly relevant to our *in vivo* MRS experiments since mice in our animal facility are routinely provided local tap water to drink. Deuterium oxide (“heavy water”; D_2_O, 99.9%; D = ^2^H; Sigma Aldrich Co.; St. Louis, MO) was mixed with tap water to produce individual samples of increasing ^2^H concentration. Because of rapid chemical exchange, ^2^H exists in these samples as ^1^HO^2^H (= HOD; “semiheavy water”), and ^2^H content was titrated in terms of ΔHOD relative to tap water. Here, ΔHOD represents ^2^H content above natural-abundance levels found in local tap water. Five ^2^H-enriched samples, having ΔHOD = 36, 72, 108, 144, and 180 mM, respectively, were prepared. Pulse-and-collect ^2^H MR spectra were acquired under quantitative conditions: 80 µs 90° rectangular RF pulse, TR 3,000 ms, 64 averages, 1,024 complex data points, and 1,500-Hz acquisition bandwidth.

#### Single Voxel Spectroscopy With Phantom

In anticipation of using the localized SPin-ECho full-Intensity Acquired Localized (SPECIAL) spectroscopy sequence (21) for *in vivo* studies, one-dimensional slice profiles in the x-, y-, and z-directions were collected with SPECIAL. Hyperbolic secant 180° and 90° adiabatic pulses were employed in concert with standard outer volume suppression (OVS) (22). The phantom consisted of a 10% D_2_O solution contained in a 5-ml plastic syringe (~1.2-mm ID). SPECIAL parameters include TR 450 ms, TE 4.27 ms, 64 averages, 512 complex data points, 2,000-Hz acquisition bandwidth, voxel size 4 × 4 × 4 mm^3^, and 10-mm OVS bands employing 90° hypersecant pulses adjacent to the edges of the single voxel on all sides.

#### Single Voxel Spectroscopy *In Vivo*

For *in vivo* experiments, the acquisition of anatomic ^1^H MRI was followed by ^1^H B_0_ map-based gradient shimming, resulting in a typical ^1^H water linewidth of ~40 Hz across a 3 × 3 × 3 mm^3^ to 4 × 4 × 4 mm^3^ voxel (27–64 µl). In tumor-bearing and RN mice, anatomic images were acquired after IP injection of a Gd-based T1 contrast agent. The voxel position of the region of interest was selected by analyzing a ^1^H multi-slice T1W image collected with a Fast Spin-Echo (FSE) sequence: TR 500 ms, TE 9.5 ms, FOV 16 × 16 mm^2^, 16 averages, slice thickness 1 mm, and matrix size 128 × 128. Localized ^2^H spectra were then acquired from a voxel positioned around the lesion region using the SPECIAL sequence with standard OVS (22) employing 90° hypersecant pulses. The OVS bands were 10-mm wide, and the suppression volume was adjacent to the edges of the single voxel on all sides. As ^2^H T2 is limited for tissue water (~20 ms) and glucose (~30 ms) (23), TE is short (4.27 ms), a desirable attribute of the SPECIAL sequence. ^2^H MRS datasets were acquired in nominal ten-minute time blocks (10.5 min). Acquisition parameters include TR 450 ms, TE 4.27 ms, 1,400 averages, 1,024 complex data points, 1,500-Hz acquisition bandwidth, voxel size 3 × 3 × 3 mm^3^ to 4 × 4 × 4 mm^3^, hyperbolic secant 180° and 90° adiabatic pulses.

#### ^2^H MRS Metabolic Monitoring

Single-voxel spectroscopy data were acquired as described above to monitor the metabolism of [6,6-^2^H_2_]glucose (Glc) for tumor-bearing, RN, and control mice (n = 4, each). Glc was injected at a dose of ~2 g/kg body weight. Dosage was not adjusted for animal weights, which varied by less than 5% across the twelve mice (mean body weight = 19.5 g; SD = 0.76 g). Before ^2^H MRS data acquisition, voxel-specific ^1^H MR magnetic-field shimming yielded water ^1^H linewidths of ~40–45 Hz (~0.08–0.09 ppm). SPECIAL ^2^H MRS data were acquired in repeated 10-min signal-averaged blocks from voxels positioned around the tumor or RN lesion. Two initial 10-min signal blocks were acquired before the administration of Glc to quantify the natural abundance of HOD signal, a convenient internal concentration reference. During the third 10-min acquisition block, 36-mg (2 × 10^−4^ mole) of Glc in 200 µl saline (180 mg/ml) was administered *via* the tail vein, and this same Glc solution was slowly infused (130 µl/h) during eight subsequent 10-min acquisition blocks. The repetitive 10-min acquisitions enabled time-course monitoring of the conversion of Glc to, ideally, [3,3-^2^H_2_]-labeled lactate (Lac) and [4,4-^2^H_2_]-labeled glutamine and glutamate, detected as the content sum of the two metabolites (Glx) (23). Based on recent ^2^H label-loss data (24), the Lac resonance was assigned a stoichiometry of 1.7 (15% loss of label) and the Glx resonance a stoichiometry of 1.2 (40% loss of label). The concentration ratio Glx : Lac provides a measure of the oxidative (TCA cycle) *vs.* glycolytic (Warburg effect) pathway flux of administered Glc.

### Hematoxylin and Eosin (H&E) Histology

Mice were sacrificed and their brains were immediately removed from the skulls and immersed in 10% formalin for 24 h. The brains were then transferred to a 20% alcohol solution. A 3-mm thick transaxial block, centered at the irradiation site (~3 mm behind the bregma), was obtained from each brain. The blocks were then processed through graded alcohols and embedded in paraffin. All paraffin-fixed blocks were sectioned from the center at a thickness of five microns. Tissue sections were stained with H&E according to standard protocols.

### Data Analysis and Statistics

#### ^1^H MRI Pipeline Analysis Strategy

All images were processed with an “un-ringing” algorithm (25) and a Gaussian filter (σ = 0.75). The R1, R2, and ADC parametric maps were computed on a voxel-wise basis with the Bayesian Toolbox (26), a data modeling software package based upon the precepts of Bayesian probability theory. The voxel-wise maps of MTR and DCE_AUC_ were calculated in MATLAB (The Mathworks, Natick, MA). The signal models/equations used in these analyses are summarized below.

For each mouse, lesions were segmented using ITK-SNAP (www.itksnap.org) (27). Regions of interest (ROI) were drawn manually based principally upon MTR and post-contrast R1 parametric maps, supported by central-lesion H&E-stained tissue slice(s). Individual ROIs were drawn on each image slice to faithfully outline regions dominated by tumor/lesion contrasts. The ROIs were drawn conservatively to avoid ventricles and partial volume effects. For RN, post-contrast R1 was the primary contrast used for segmentation; for tumors, MTR and post-contrast R1 were used iteratively for segmentation.

#### MR Signal Models

Data were analyzed using standard signal models, as described below.

- The R1 maps were calculated *via* a variable flip angle (θ) experiment S(θ) = S(Boltzmann) × (1 − exp(−TR × R1)) × sin(θ)/(1 − exp(−TR × R1) × cos(θ)). [1]

- The R2 maps were calculated *via* a multi-spin-echo (i.e., multi-TE) experiment: S(TE) = S(TE = 0) × exp(−TE × R2) + Constant.[2]

- ADC maps—1/3 × Trace of the diffusion tensor D (ADC = 1/3 × Trace(D))—were calculated from diffusion-weighted MR images *via* six diffusion-encoding b-vectors and a b = 0 acquisition, accounting for the diffusion weighting of the imaging gradients:

S(b) = S(b = 0) × exp(−b × D),
  in which b is formally the b-matrix for each diffusion-weighted MR image.[3]

- MTR maps were calculated *via* the normalized ratio of signal difference from experiments with MT RF irradiation (ON) and without MT RF irradiation (OFF): MTR = 100% × (OFF − ON)/OFF.[4]

- The area under the DCE time-course curve (DCE_AUC_) was calculated following contrast-agent injection. To account for baseline (pre-administration of contrast agent) signal variations between subjects, the signal at each time point was expressed as the fractional enhancement in voxel intensity relative to the pre-contrast period. The signal was further normalized by the maximum fractional enhancement in the early time frames (nos. 31–120) of the temporalis and masseter muscles to account for potential modest variations in contrast-agent delivery. AUC was expressed on a per-unit-time (s) basis by dividing the sum of the normalized signal over the post-injection time frames by 810 (3 s/frame × 270 post-injection frames).

#### ^2^H MRS Analysis

^2^H FID’s were analyzed *via* Bayesian time-domain signal modeling as sums of exponentially decaying sinusoids using the Bayesian Toolbox (26). Bayesian modeling provides probability density functions (PDFs) for each resonance frequency, decay-rate constant (R2*), and amplitude. Estimates of these parameter values were assigned as the means of each parameter’s PDF; uncertainties were assigned as the standard deviation of that parameter’s PDF. For each resonance, the frequency and R2* were estimated as common values across a given time course. Individual resonance amplitudes were estimated for each time frame. The ^2^H resonance amplitudes for Glc, Lac, and Glx, proportional to metabolite concentrations, were corrected for labeling stoichiometry (*vide supra*), and relaxation effects using published T1 and T2 values (23). Amplitudes of the modeled water (HOD) and metabolite ^2^H resonances were converted to concentrations (mmoles/liter of tissue water) using the amplitude of the natural-abundance HOD signal acquired before administration of Glc as an internal reference.

### Statistical Analysis

Differences in measured ^1^H MR parameters (e.g., R1, R2, ADC, MTR, DCE_AUC_) between pure tumor and pure RN, and differences between different models (e.g., tumor in the mixed lesion model vs. pure tumor; necrosis in the mixed lesion model *vs.* pure necrosis) were compared using two-tailed t-tests. Parameter values for tumor and necrosis within the mixed lesion model were compared using a paired t-test. The resultant p-values were adjusted by a step-down Bonferroni adjustment for multiple comparisons. Time-course ^2^H spectroscopy data were analyzed using two-way ANOVA for repeated measurement, followed by *post-hoc* comparison of the least squares means across groups (control vs. tumor-bearing vs. necrosis). All analyses were performed using SAS 9.4 (SAS Institutes, Cary, NC). Statistical significance was defined as a two-tailed p-value of <0.05.

## Results

### The Mixed Tumor/Radiation Necrosis (RN) Model

**Figure 2:**
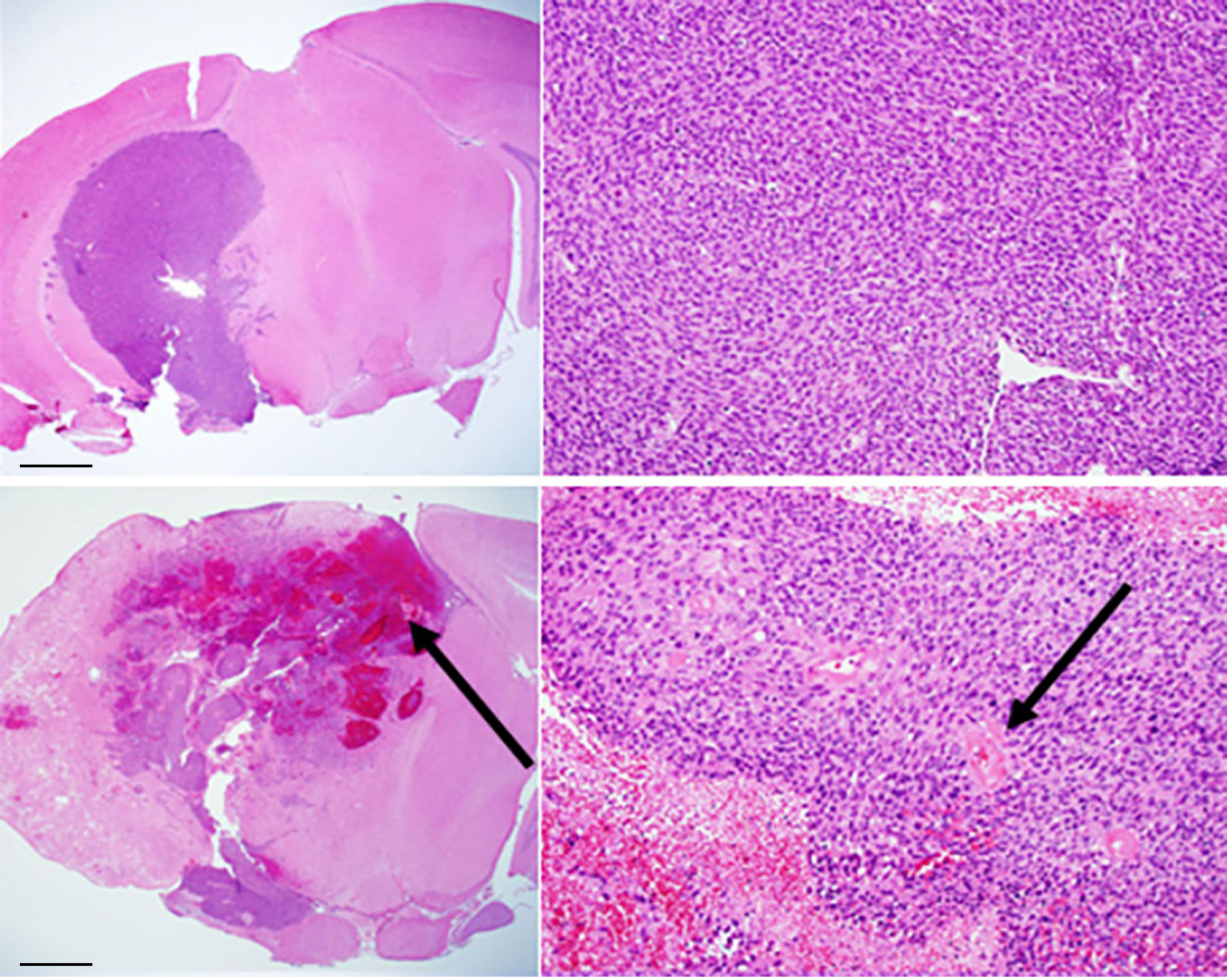
Conventional H&E staining of: (top) GL261 tumor growing in non-irradiated mouse brain (C57BL/6); (bottom) mixed model, with tumor growing within a region of radiation necrosis. Scale bars on the whole-mount images (left) represent a length of 1 mm; the expanded images (right) are at a magnification factor of 10×. Black arrows indicate areas of hemorrhage and necrosis (bottom, left) and vasculopathic changes (bottom, right).

As noted earlier, three preclinical models, namely, orthotopically implanted tumor, pure RN, and the mixed tumor/RN model, are well established in our lab (9–18). In the mixed tumor/RN model, GL261 tumor cells were implanted into the previously irradiated brain four weeks post-irradiation. Conventional H&E staining of the tumor in the non-irradiated brain, **Figure 2** (top), shows a relatively solid malignant glioma in the brain. High-grade gliomas are characterized by dense cellularity, significant pleomorphism, and frequent mitoses. By contrast, in the mixed model, **Figure 2** (bottom), malignant glioma cells are admixed within a region of RN; the latter is characterized by areas of rarefaction, gliosis, and vasculopathy obvious within the brain parenchyma surrounding the implanted malignant tumor cells. Also notable is the presence of necrosis within the tumor itself, which was much more frequent in this group compared to mice implanted with tumor alone (i.e., without RN in the background).

### ^1^H MRI Pipeline

**Figure 3:**
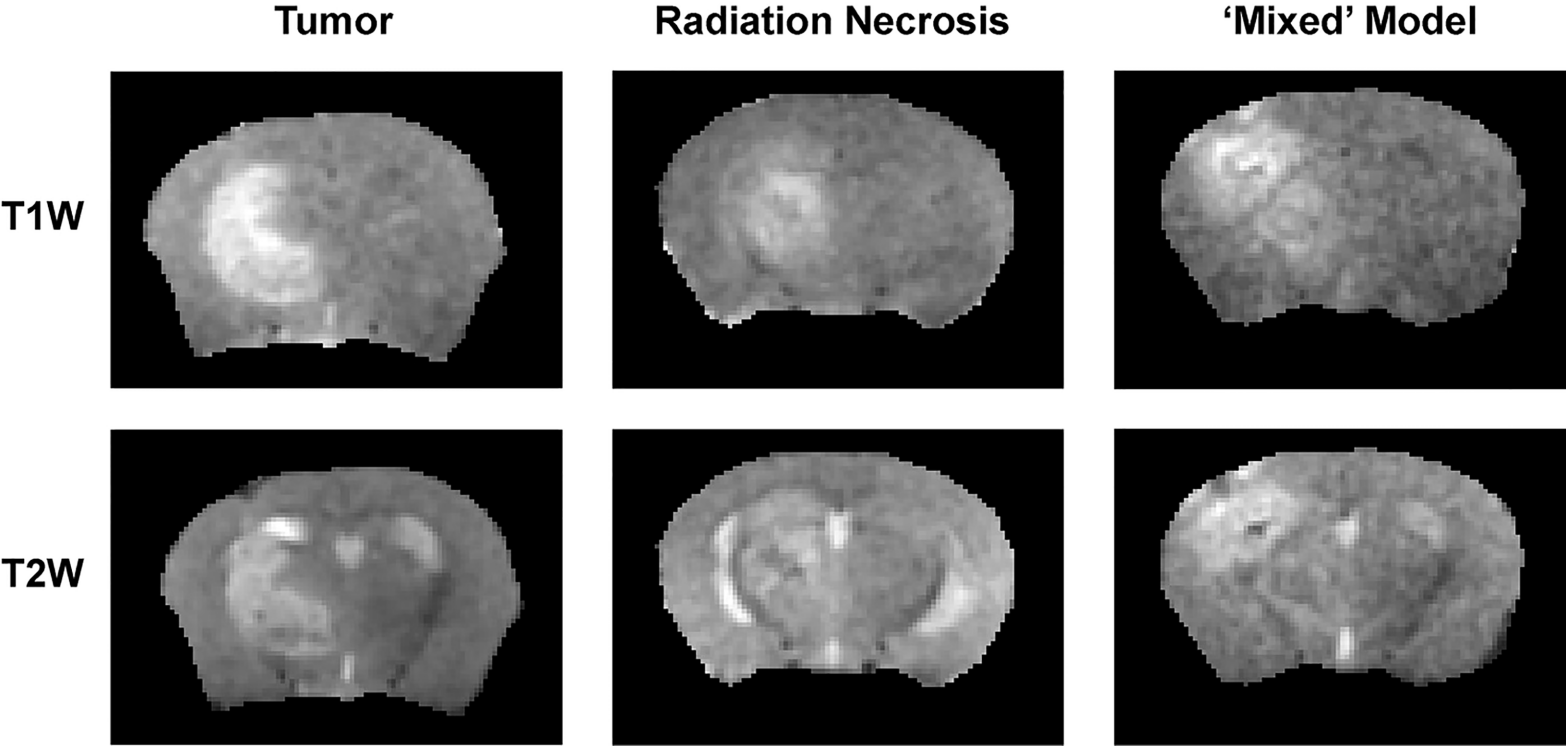
(top) T1-weighted and (bottom) T2-weighted images, both acquired post contrast, of: (left) pure tumor; (middle) pure RN; and (right) the mixed model.

**Figure 4:**
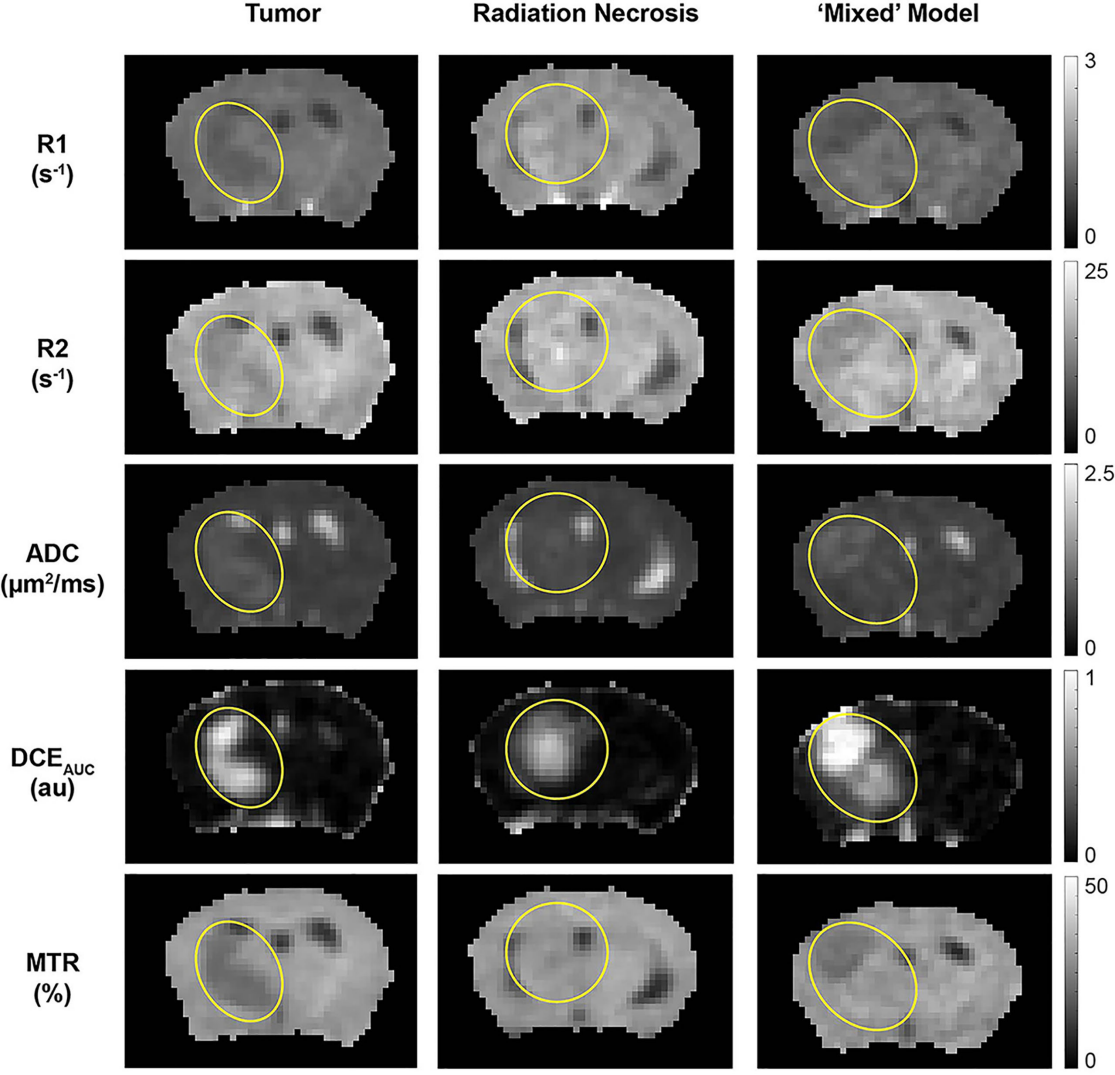
Parametric maps, derived from the ^1^H MRI pipeline of (left) pure tumor; (middle) pure radiation necrosis, and (right) mixed model. From top to bottom, maps are shown of R1 (= 1/T1), R2 (= 1/T2), apparent diffusion coefficient (ADC), DCE area under the curve (AUC), and magnetization transfer ratio (MTR). Yellow circles/ovals guide the eye to the locations of lesions in each of these maps.

The ^1^H MR pipeline (**Figure 1**) was applied to study three different cohorts of mice—pure RN, pure tumor, and mixed RN/tumor—with the goal of identifying individual imaging contrasts, or combinations thereof, for distinguishing pure RN from RN admixed with tumor. Representative post-contrast T1W and T2W images of the brains of mice in each of these cohorts are shown in **Figure 3**. Lesions are clearly visible in each of these images, and total lesion volumes (RN, tumor, mixed tumor + RN) were measured quantitatively by inspection of T1W and T2W images and manual segmentation. However, anatomic images alone are not able to reliably distinguish between RN and tumor. To address this challenge, we computed parametric maps of R1, R2, ADC, DCE_AUC_, and MTR. Representative parametric maps illustrating typical data quality are shown in **Figure 4**. Yellow circles/ovals drawn on these maps guide the eye to the locations of lesions. Regions of interest corresponding to lesions observed in T1W and T2W images were drawn manually on each parametric map. **Table 1** summarizes measurements of R1, R2, ADC, DCE_AUC_, and MTR in pure tumor, pure RN, and the mixed tumor/RN model. For each member of the mixed model cohort, regions of confirmed tumor and of RN were identified by histology, allowing regions of interest for tumor and for RN to be drawn separately on the various parametric maps. Thus, separate mean parameter values (± SD) for tumor and for RN in the mixed model are reported in the table (lines 3 and 4, respectively). [Note: While the variable flip angle (VFA) R1 mapping method used clinically is time-efficient, results are confounded by a substantial MT effect. Compared to single-slice, “gold standard” inversion recovery (IR) R1 mapping, having minimal MT effect, the lesion VFA R1 values were 1.8× greater than the corresponding IR R1 values (data not shown)].

**Table 1:** Mean MR Parameter Values.

	R1 (SD) [s^−1^]	R2 (SD) [s^−1^]	ADC (SD) [μm^2^/ms]	AUC (SD) [au]	MTR (SD) [%]
Tumor	1.08 (0.11)	14.9 (0.87)	0.833 (0.053)	0.916 (0.256)	24.1 (1.5)
RN	1.26 (0.09)	17.6 (0.72)	0.736 (0.052)	0.784 (0.247)	30.2 (1.5)
Tumor in Mixed	1.05 (0.06)	14.2 (0.59)	0.872 (0.042)	1.22 (0.37)	22.2 (0.9)
RN in Mixed	1.28 (0.08)	17.7 (1.16)	0.704 (0.049)	0.658 (0.110)	29.0 (1.6)

The quantitative MR parametric values of R1, R2, ADC, and MTR comparing tumor alone (n = 9) *vs.* RN alone (n = 9) are all highly statistically significant (**Table 2**). Differences in these parameter values, as well as DCE_AUC_, for tumor *vs.* RN in the mixed model are also all significant, demonstrating that these contrasts can all effectively distinguish tumor from RN in the mixed model. Readouts from the pure models (tumor alone, RN alone) were largely similar (i.e., not statistically different) compared to regions of the same lesion in the mixed model. Only *tumor* MTR and DCE_AUC_ values showed statistically significant differences comparing pure tumor *vs.* mixed-lesion tumor (p = 0.013 and p= 0.035, respectively), and these were modest compared to differences in MTR and DCE_AUC_ metrics between mixed-lesion tumor *vs.* mixed-lesion RN. Thus, the pure lesion models provide substantial predictive insight into MRI parameter readouts that are characteristic of tumor *vs.* RN regions in the mixed lesion. An additional feature of these mouse models is that cortex in the contralateral brain-hemisphere provides intra- and inter-subject non-lesion control tissue. Indeed, comparing each ^1^H MRI parameter for the contralateral cortex across the three lesion model cohorts (tumor alone, RN alone, and mixed), none showed statistically significant differences, except for MTR comparing contralateral cortex in the mixed lesion *vs.* RN alone models (p = 0.01), and the difference was small (5%).

**Table 2:** Statistical Comparison (p-values) of MR Parameter Values.

	R1	R2	ADC	AUC	MTR
Tumor vs. Tumor in Mixed Model	0.414	0.138	0.142	**0.0348**	**0.0134**
RN vs. RN in Mixed Model	0.615	0.786	0.224	0.362	0.0913
Tumor vs. RN	**0.0007**	**<.0001**	**<.0001**	0.326	**<.0001**
Tumor in Mixed vs. RN in Mixed Model	**<.0001**	**<.0001**	**0.0005**	**0.0004**	**<.0001**

Bold values indicates statistical significance at p < 0.05 level.

**Figure 5:**
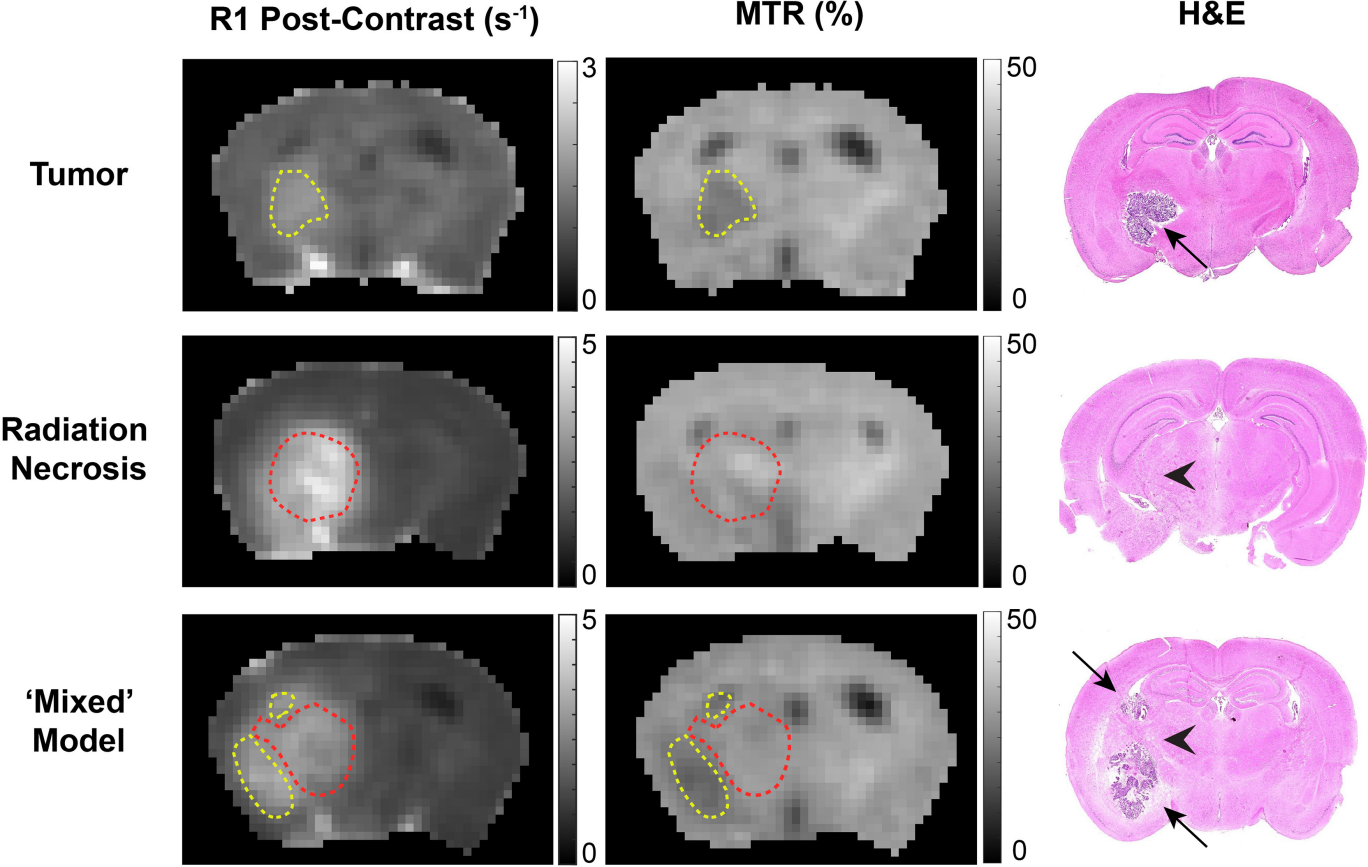
Contrast-enhanced (left) T1-weighted images, (middle) magnetization transfer ratio (MTR) parametric maps, and (right) H&E staining of: (top) pure tumor; (middle) pure radiation necrosis; (bottom) mixed model, with tumor growing within a region of radiation necrosis. Red contours outline regions-of-interest of radiation necrosis; yellow contours outline regions-of-interest of tumor. In the H&E, black arrows identify tumor and black arrow heads identify RN.

Of particular note is our observation that MTR cleanly discriminates between tumors and RN. Distinct from an earlier MTR protocol reported by our lab (14), MTR pulse sequence parameters were chosen to avoid substantial direct T1 weighting (TR = 2 s) and to minimize direct saturation of the water resonance (~4%). The ability of MTR contrast to distinguish tumor *vs.* RN in the mixed lesions is illustrated in **Figure 5**. Here, post-contrast T1W images, MTR parametric maps, and H&E histology are shown for pure tumor, pure RN, and the mixed model. In this figure, red contours outline ROIs of RN; yellow contours outline ROIs of the tumor. The location of the tumor and/or RN in the T1W images and MTR parametric maps is confirmed by histology. Consistent with the numbers reported in **Table 1**, tumors appear dark on maps of MTR, while RN lesions are indistinguishable from the contralateral cortex. Further, MTR of the pure RN lesion is not statistically different from RN in the mixed lesion. Thus, once lesions are identified in anatomic images, MTR serves to cleanly distinguish tumors from RN.

### ^2^H MRS

#### Semiheavy Water Natural Abundance (St. Louis Region)

**Figure 6:**
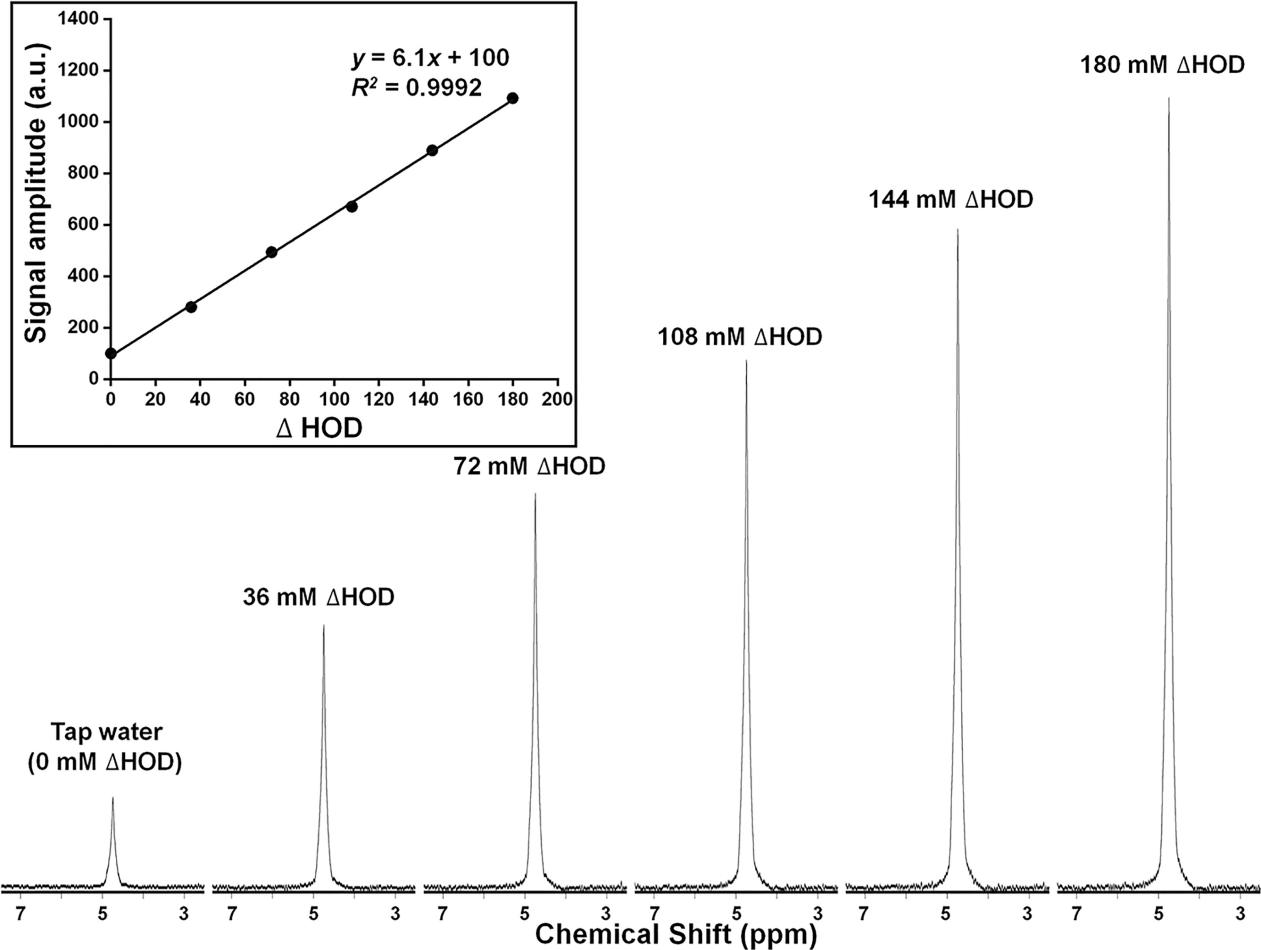
Deuterium (^2^H = D) MR spectra and (inset) plot of signal amplitude (arbitrary units), for a series of HOD samples of increasing ^2^H concentration. ^2^H concentration (ΔHOD) is expressed relative to that of tap water.

Titration experiments were performed on a series of water phantoms of increasing deuterium concentration, added as D_2_O. As noted earlier, because of rapid exchange, the actual deuterated species present in these samples is principally HOD. For simplicity, we express the composition of these phantoms in terms of ΔHOD, in which ΔHOD represents ^2^H content above natural-abundance levels found in local tap water. **Figure 6** shows ^2^H MR spectra acquired under quantitative pulse-and-collect conditions. The spectra are characterized by high signal-to-noise and symmetric lineshape. The linearity of detected signal amplitude with ^2^H concentrations ranging from ΔHOD = 0 mM to ΔHOD = 180 mM is illustrated in the inset plot in this figure. From these data, the tap water natural-abundance ^2^H MR signal amplitude corresponds to an HOD concentration of 16.35 mM.

#### Single Voxel (SPECIAL) Profiles

**Figure 7:**
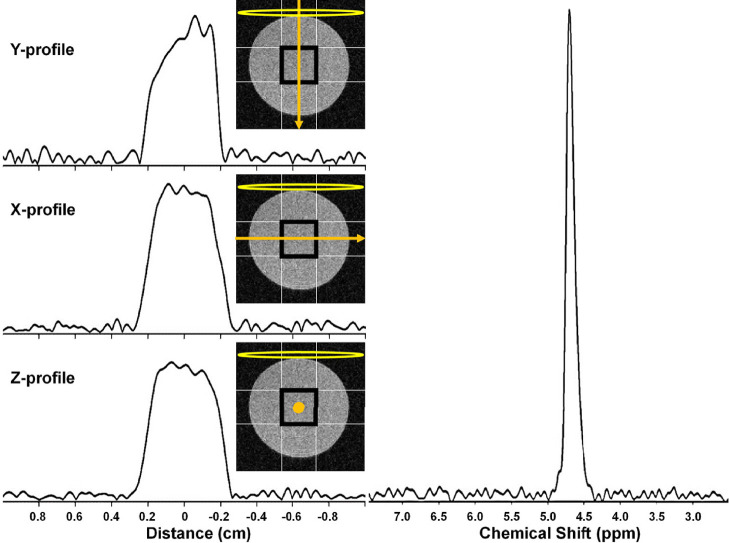
(left) ^2^H MRI profiles from a 4 × 4 × 4 mm^3^ SPECIAL voxel in a 10% D_2_O cylindrical phantom in the (top) Y, (middle) X, and (bottom) Z. The nominal selected voxel is shown as a black square overlaid on a transverse slice (light gray) through the phantom, the profile direction as an orange arrow, and the RF surface coil as a yellow oval. (right) ^2^H MR spectrum resulting from selection of this 4 × 4 × 4 mm^3^ voxel.

**Figure 7** shows x-, y-, and z-direction ^2^H MRI profiles for a 4 × 4 × 4 mm^3^ SPECIAL voxel in the 10% D_2_O phantom. The nominal selected voxel is shown as a black square in this figure, the profile direction as an orange arrow, and the RF surface coil as a yellow oval. Symmetric, rectangular profiles are observed in the x and z directions, perpendicular to the applied RF field. The profile in the y-direction is less symmetric, reflecting the drop-off in RF receptivity with increasing distance from the surface coil. The ^2^H MR spectrum resulting from the selection of this voxel is shown in **Figure 7**.

#### ^2^H MRS Metabolic Monitoring

**Figure 8:**
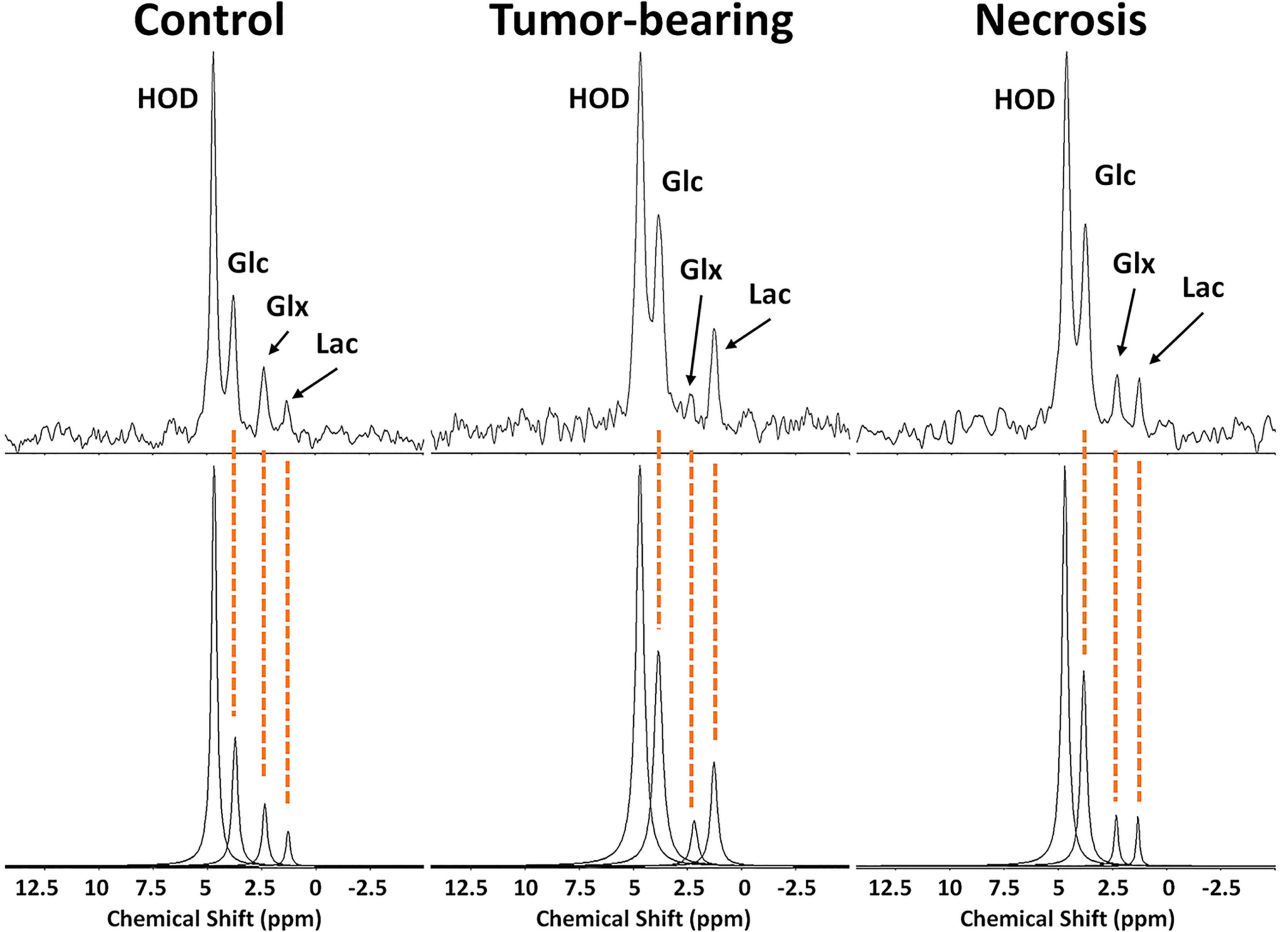
(top) ^2^H MR spectra created by summation of nine 10-min acquisitions during administration of Glc for (left) control, non-lesion-bearing mouse; (middle) pure tumor; (right) pure radiation necrosis. (bottom) Results of Bayesian modeling of these spectra. Estimates and uncertainties of resonance frequencies, amplitudes, and linewidths derived from decay-rate constants (R2*) for these spectra are summarized in **Supplementary Data, Table S1**.

**Figure 9:**
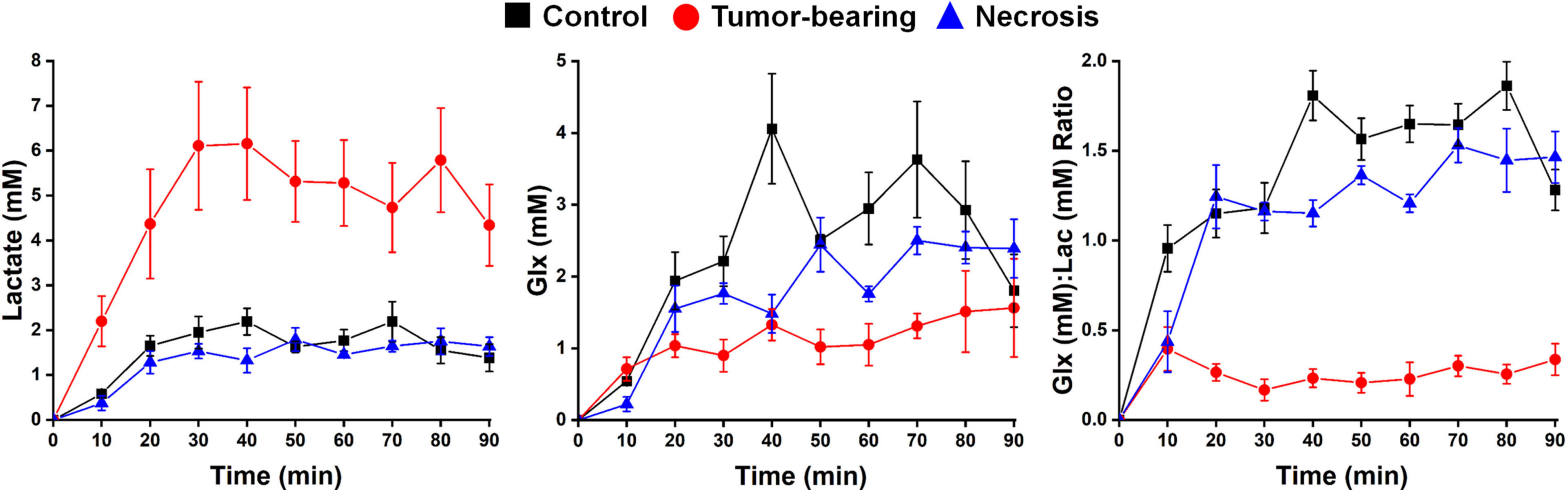
Time-course plots (means ± SEM) for the concentration of (left) Lac and (middle) Glx, and (right) the concentration ratio Glx : Lac derived from a 90-min series of 10-min acquisition spectra with n = 4 mice each condition (control, tumor-bearing, radiation necrosis). These time-course spectroscopy data were analyzed using two-way ANOVA for repeated measurement, followed by *post-hoc* comparison of the least squares means (LSM) across groups. ANOVA2/LSM results are—Lac: tumor vs. necrosis, p <0.0001; tumor vs. control, p <0.0001; necrosis vs. control, p = 0.7631; Glx: tumor vs. necrosis, p = 0.0671; tumor vs. control, p = 0.0004; necrosis vs. control, p = 0.0685; Glx/Lac: tumor vs. necrosis, p <0.0001; tumor vs. control, p <0.0001; necrosis vs. control, p <0.0001.

^2^H MRS is an emerging modality for measuring tumor metabolism in tissues, and this study focused on its ability to identify tumor and to distinguish tumor from RN. **Figure 8** shows representative ^2^H MR spectra created by the summation of the nine 10-min acquisitions during the administration of Glc. Results of Bayesian spectral modeling (“decomposition”) are also shown (Representative ^2^H MR spectra acquired from a single 30–40 min time block are shown in **Supplemental Data Figure S1**). The HOD and Glc ^2^H linewidths in the summed data were ~15–20 and 20–25 Hz, respectively (0.2–0.3 ppm). Substantially higher Lac and lower Glx signal amplitudes are seen for tumors *vs.* RN. **Figure 9** shows time-course plots for the concentration of Lac and for the concentration ratio Glx : Lac derived from these spectra, with Lac markedly higher (p <0.0001) and Glx : Lac markedly lower (p <0.0001) in tumor *vs.* RN. Compared to RN, the tumor almost exclusively converts Glc to Lac in preference to oxidative metabolism (conversion to Glx). Note the substantial dynamic range (~5×) of both Lac concentration and Glx : Lac as ^2^H MRS-derived biomarkers for distinguishing tumors and RN.

## Discussion

### MR Indices of Tumor vs. RN

While most patients treated for malignant brain tumors are followed during and after treatment with serial MR scans to assess response to therapy and tumor stability, to date there is no clinical MRI (or positron emission tomography (PET)) protocol that consistently and accurately distinguishes pure RN from mixed recurrent tumor/RN (4). Investigators have attempted to resolve this clinical problem by combining i) anatomic information from CE T1W and T2W MRI; ii) microstructural information from diffusion-weighted MRI; iii) functional information from perfusion studies, including DCE or dynamic susceptibility contrast (DSC) perfusion MRI; and iv) metabolic information from ^1^H MRS. Although large meta-analyses of multiparametric MRI studies designed to assess the differentiation of RN from recurrent malignant tumors/RN showed that perfusion weighted imaging metrics were relatively good at differentiation, significant variability in reported optimal thresholds and other limitations have challenged widespread quantitative diagnosis (2). An important shortcoming in the use of DSC-MRI is that its cerebral blood volume-based tumor signature can be unavailable/unreliable in some patients secondary to susceptibility artifacts from nearby bone, calcification, or blood related to microhemorrhages, or due to overlapping low CBV values in both conditions, i.e., RN *vs* recurrent tumor/RN. Indeed, while CBV-based tumor signatures have been shown to correlate histologically with image-guided biopsies in a majority of specimens, up to 15% failed to distinguish lesion identity secondary to technical problems (1), or were inconclusive as a result of overlapping lower thresholds (20). Similar indeterminacy is evident from ^1^H MRS studies (5–8) in which various endogenous metabolite metrics show 70–80% sensitivity and specificity in distinguishing RN *vs.* recurrent tumor. For example, in a study of 23 patients, DSC combined with MRS was diagnostically successful in only 18 patients (72%) (28). The above studies identify a continued need for improved, widely available imaging biomarkers to distinguish pure RN from recurrent tumors admixed in RN.

Reprogramming of glucose uptake and subsequent glycolysis, a hallmark of malignant cancer cells and distinct from normal cells, was initially described as the Warburg effect (29, 30). It is a strategic metabolic switch in glucose metabolism from predominantly oxidative phosphorylation in normal cells to altered uptake and metabolism of glucose in actively proliferating malignant cells. Under this altered metabolism, glucose is avidly taken up by the tumor cells and the majority is metabolized to lactate (fermentation) that is subsequently secreted from the cells. This process is known as aerobic glycolysis as it occurs despite the presence of sufficient oxygen to support respiration. Aerobic glycolysis is utilized by actively proliferating tumor cells having a metabolic phenotype distinct from normal cells. Indeed, tumor cells exhibit glycolytic flux at rates up to 200 times those of normal cells. Aerobic glycolysis generates adenosine triphosphate for energy and increases the activity in the pentose phosphate pathway. The potential utility of aerobic glycolysis as an important metabolic bio-marker of tumor growth and aggressiveness in high-grade gliomas was identified in patients undergoing both [2-^18^F]FDG and [^15^O]-labeled CO, O_2_ and H_2_O PET studies, with the calculation of a glycolytic index defined by linear regression of the cerebral metabolic rates of glucose and oxygen (31). The identification of elevated aerobic glycolysis as a biomarker of tumor aggressiveness was correlated with a poor prognosis in patients with high-grade gliomas (29).

An attractive alternative to PET, ^2^H MRS can quantify the Warburg effect, thereby providing a tumor biomarker. ^2^H MR takes advantage of rapid (quadrupolar dominated) spin-lattice relaxation to efficiently time average signals. Scanning times for ^2^H MRS are generally quite long, e.g., tens of minutes, and while human ^2^H MR studies have been reported at 4 tesla (23) and are certainly feasible at 3 tesla, signal-to-noise benefits substantially from high magnetic field strength. ^2^H MRS, also referred to as deuterium metabolic imaging DMI (23, 32–42), monitors the metabolic products of administered deuterated substrates. ^2^H MRS monitoring of the glycolytic conversion of Glc to Lac has shown the tumor-signature Warburg effect in rat (23) and mouse (40) glioblastoma models and clinically in human patients with malignant gliomas (23). Inhibited glycolytic conversion of Glc to Lac following chemotherapy (etoposide) was demonstrated by ^2^H MRS in a mouse lymphoma tumor model (35).

Here, we determined the utility of various ^1^H MRI contrasts that are readily/routinely available in the clinic for distinguishing malignant gliomas from RN in three cohorts of mice: malignant glioma; pure RN; and in the mixed lesion (malignant glioma growing on an RN background). Our ^1^H MRI pipeline includes parametric maps of R1, R2, ADC, DCE_AUC_, and MTR, and the results in **Table 1** clearly demonstrate the discriminating power of these contrasts. Of particular interest and novelty is the success of magnetization transfer (MT) contrast (**Figure 4**) for distinguishing tumors and RN. Previously, MT has been proposed in a limited number of studies. Van Zijl described a related approach, chemical-exchange saturation transfer (CEST), specifically, amide proton exchange (APT), for distinguishing RN *vs.* tumor (43–45). Recently, Mehrabian et al. (46), evaluated various CEST metrics in a group of patients with prior stereotactic radiosurgery (SRS) for metastatic brain disease and found that while APT did not distinguish RN from progressive disease, various MTR-related indices did. Acquiring quantitative CEST measures requires considerable care in data acquisition and preprocessing. Our MTR approach is simple, robust, and necessitates minimal data processing.

In concert with the ^1^H MRI pipeline, we also validated ^2^H MRS as a modality for identifying tumors and distinguishing them from RN. Using Glc as the administered metabolic substrate, we demonstrated that Lac production is dramatically elevated and Glx production is significantly reduced in tumor *vs.* control brain tissue. These findings are consistent with markedly increased aerobic glycolysis (i.e., Warburg effect, fermentation) and reduced oxidative glucose metabolism (respiration) in tumors. In contrast, Lac and Glx levels are only slightly changed in RN vs. control, thus resembling more closely the oxidative glucose metabolism observed in normal brain than in tumor.

### Experimental Considerations

A useful attribute of ^2^H MR *in vivo* is that the natural abundance of HOD ^2^H signaling provides an internal concentration reference. Bowen et al. have assessed the stable isotope ratios of tap water in the contiguous United States (20). ^2^H in tap water in the St. Louis, Missouri region was found to vary between 145.9 and 148.3 ppm. Given that water is 111.1 M in equivalent ^1^H, this translates to a concentration range of 16.21–16.47 mM, with an average of 16.34 mM. Our standard addition MR titration results were strongly confirmatory, yielding an HOD concentration of 16.35 mM.

Several previous ^2^H MRS studies have used chemical shift imaging (CSI) techniques to select a region of interest and generate a grid of voxels to cover that region. In contrast, the ^2^H MRS experiments described here were all single-voxel spectroscopy measurements, SPECIAL with OVS. A challenge for CSI is the significant point-spread function associated with the method, which introduces uncertainty as to exactly where the observed signals originate. A challenge with the single-voxel spectroscopy measurements described here is the use of a surface RF coil as both transmitter and receiver, i.e., in the single-coil mode. Surface coils are well understood to present an inhomogeneous RF magnetic field (B_1_) whose amplitude is strongest near the plane of the coil but decreases substantially away from the coil plane. Surface coils provide a sensitive volume nominally characterized by the coil radius, with coils of smaller radii providing increased sensitivity, but over a more limited volume. We chose a ~2-cm coil ID as a trade-off between maximum sensitivity and full brain coverage as qualitatively assessed by ^2^H CSI experiments with concentrated D_2_O phantoms (data not shown).

To confirm the ^2^H MRS performance of the SPECIAL pulse sequence with surface-coil driven adiabatic 180° and 90° pulses, x, y, and z projections of the single-voxel profile were obtained with a phantom (**Figure 7**). The voxel dimensions were well-defined with sharp boundaries. As expected, the profile orthogonal to the coil plane displays the well-known decreasing sensitivity with increasing depth away from the coil. “Contaminating” signals arising from regions outside the SPECIAL voxel are minimal. (Recall that OVS was also employed in concert with the SPECIAL pulse sequence). Following initial T1W ^1^H scans to define a given lesion’s position and volume, the SPECIAL voxel position, size, and orientation were nominally customized to fit the lesion. Here, the 6.5× difference in ^1^H *vs.* ^2^H resonance frequencies, 500 *vs.* 76.6 MHz, coupled with the 2.5× difference in coil dimensions, 5-cm ^1^H volume coil *vs*. 2-cm ^2^H surface coil, advantageously led to near zero coupling between the ^1^H and ^2^H channels. Thus, active or geometrical decoupling of ^1^H *vs.* ^2^H RF circuits was not required, a considerable experimental simplification.

Finally, we note that the SPECIAL single-voxel ^2^H spectroscopy pulse sequence employed a short (4.27 ms) but finite TE, which could hamper the detection of lower abundance ^2^H-labeled metabolites compared to ^2^H MRS pulse-and-acquire approaches, as employed recently in normal rodent brain (36) and GL261 mouse tumors (40).

### Summary

Employing mouse models of glioblastoma (GL261) and (Gamma Knife-induced) radiation necrosis, we have shown that a ^1^H MRI pipeline of commonly implemented protocols—maps of R1, R2, ADC, MTR, and DCE_AUC_—provides substantial discriminatory power to differentiate tumor from RN in both pure and mixed-lesion cases. MTR scanning with long TR and minimal direct saturation was particularly promising in this regard. Leveraging the potential of MTR scanning as a complementary imaging metric within a clinical MRI pipeline may improve diagnostic capability in staging patients for recurrent brain tumors.

Additionally, the application of ^2^H MRS, in concert with the infusion of Glc, provided substantial dynamic range in distinguishing the two pure lesions, as tumors showed a strong Warburg effect (aerobic glycolysis; fermentation) *vs.* oxidative glucose metabolism (respiration) in RN and in control, normal brain. These findings suggest a role for ^2^H metabolic imaging as a possible means for substantially improving the discrimination and staging of tumors *vs*. RN in the clinic.

## Data Availability Statement

The raw data supporting the conclusions of this article will be made available by the authors, without undue reservation.

## Ethics Statement

The animal study was reviewed and approved by the Institutional Animal Care and Use Committee, Washington University in Saint Louis.

## Author Contributions

Conceptualization/Study Design: JG, KR, and JA. Data acquisition, analysis, and curation: XG, KS, JE, LY, and SD. Statistical analysis: FG. Writing—original draft: JG and JA. Writing—review and editing: JG, JA, KR, XG, and KS. All authors listed have made a substantial, direct, and intellectual contribution to the work and approved it for publication.

## Funding

This research was funded by the Alvin J. Siteman Cancer Center Investment Program (supported by the Foundation of Barnes- Jewish Hospital, Cancer Frontier Fund; National Cancer Institute, Cancer Center Support Grant, P30 CA091842; and Barnard Trust). The studies presented in this work were carried out, in part, using the Small Animal MR Facility of the Mallinckrodt Institute of Radiology, with support from the Small-Animal Cancer Imaging Shared Resource of the Siteman Cancer Center.

## Conflict of Interest

The authors declare that the research was conducted in the absence of any commercial or financial relationships that could be construed as a potential conflict of interest.

## Publisher’s Note

All claims expressed in this article are solely those of the authors and do not necessarily represent those of their affiliated organizations, or those of the publisher, the editors and the reviewers. Any product that may be evaluated in this article, or claim that may be made by its manufacturer, is not guaranteed or endorsed by the publisher.
