# Supplementary figures and images for: Distinguishing Tumor Admixed in a Radiation Necrosis (RN) Background: 1H and 2H MR With a Novel Mouse Brain-Tumor/RN Model
Source: Front Oncol. 2022 May 30;12:885480. doi: 10.3389/fonc.2022.885480 (PMC9196939; doi:10.3389/fonc.2022.885480)

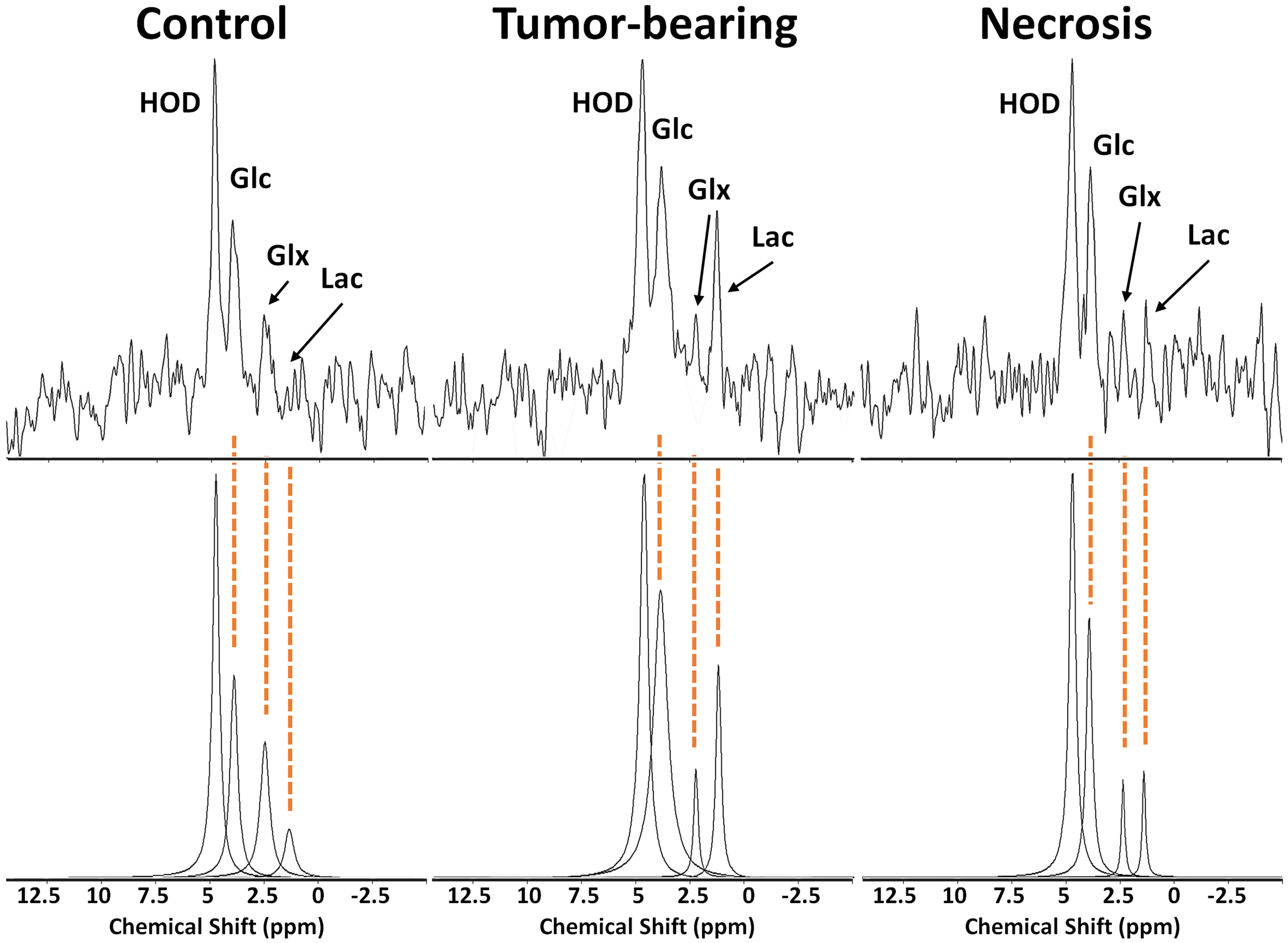

Supplement: Supplementary file 1 [file Image_1.jpeg]
