# Supplementary material for: Distinguishing Tumor Admixed in a Radiation Necrosis (RN) Background: 1H and 2H MR With a Novel Mouse Brain-Tumor/RN Model
Source: Front Oncol. 2022 May 30;12:885480. doi: 10.3389/fonc.2022.885480 (PMC9196939; doi:10.3389/fonc.2022.885480)
Supplement: Supplementary file 2 [file Table_1.pdf]

Supplemental Table.

Estimates and uncertainties of resonance frequencies, amplitudes, and linewidths derived from decay-rate constants (R2*), for the spectra in Figure 8 are summarized in Table S1 below.

Control	Lac		Glx		Glc		HOD	
	Mean	SD	Mean	SD	Mean	SD	Mean	SD
Frequency (ppm)	1.36	0.03	2.37	0.02	3.85	0.02	4.71	0.01
Linewidth (Hz)	11.7	3.4	12.6	3.6	17.1	2.5	19.6	1.7
Amplitude	109	38	187	40	411	44	973	39

Tumor	Lac		Glx		Glc		HOD	
	Mean	SD	Mean	SD	Mean	SD	Mean	SD
Frequency (ppm)	1.33	0.02	2.39	0.05	3.76	0.01	4.72	0.01
Linewidth (Hz)	13.3	2.9	12.4	8.9	24.4	3.5	21.2	1.2
Amplitude	368	31	58	20	752	44	1318	38

Radiation Necrosis	Lac		Glx		Glc		HOD	
	Mean	SD	Mean	SD	Mean	SD	Mean	SD
Frequency (ppm)	1.32	0.03	2.36	0.03	3.81	0.02	4.70	0.01
Linewidth (Hz)	10.6	5.3	13.1	5.4	20.6	4.1	18.5	1.3
Amplitude	111	24	123	28	385	34	858	31
